# Correction: The added value of devices to pelvic floor muscle training in radical post-prostatectomy stress urinary incontinence: A systematic review with metanalysis

**DOI:** 10.1371/journal.pone.0315959

**Published:** 2024-12-12

**Authors:** Benedetto Giardulli, Simone Battista, Gaia Leuzzi, Mirko Job, Ottavia Buccarella, Marco Testa

The author names are incorrect. The first names and last names are switched. The correct author names are: Benedetto Giardulli, Simone Battista, Gaia Leuzzi, Mirko Job, Ottavia Buccarella, Marco Testa. The correct citation is: Giardulli B, Battista S, Leuzzi G, Job M, Buccarella O, Testa M (2023) The added value of devices to pelvic floor muscle training in radical post-prostatectomy stress urinary incontinence: A systematic review with metanalysis. PLoS ONE 18(9): e0289636. https://doi.org/10.1371/journal.pone.0289636.
