# Supplementary material for: The added value of devices to pelvic floor muscle training in radical post-prostatectomy stress urinary incontinence: A systematic review with metanalysis
Source: PLoS One. 2023 Sep 28;18(9):e0289636. doi: 10.1371/journal.pone.0289636 (PMC10538711; doi:10.1371/journal.pone.0289636)
Supplement: S1 File — (DOCX) [file pone.0289636.s001.docx]

**Supplementary material 1: Database research strings**

PubMed Research String

| Population | Intervention | Comparison | Outcome |
| --- | --- | --- | --- |
| ("urinary incontinence, stress"[MeSH Terms] OR "Urinary Incontinence"[MeSH Terms] OR "Prostatectomy"[MeSH Terms]) AND "Male"[MeSH Terms] | (((((((("Physical and Rehabilitation Medicine"[Mesh]) OR ("Physical Therapy Modalities"[Mesh])) OR ("Physical Therapy Modalities"[Mesh])) OR ("Rehabilitation"[Mesh])) OR ("Physical Education and Training"[Mesh])) OR ("Exercise"[Mesh])) OR ("Pelvic Floor"[Mesh])) OR ("Feedback, Psychological"[Mesh])) OR ("Equipment and Supplies"[Mesh]) | Pelvic training alone | ((((((((("Lower Urinary Tract Symptoms"[Mesh]) OR (ICIQ-SF)) OR (Urogenital Distress Inventory)) OR (UDI)) OR (muscle strength)) OR ("Muscle Strength"[Mesh])) OR ("Muscle Strength Dynamometer"[Mesh])) OR (international consultation on incontinence questionnaire)) OR (Incontinence Impact Questionnaire)) OR (IIQ-7) |
| ("urinary incontinence, stress"[MeSH Terms] OR "Urinary Incontinence"[MeSH Terms] OR "Prostatectomy"[MeSH Terms]) AND "Male"[MeSH Terms] AND ("Lower Urinary Tract Symptoms"[MeSH Terms] OR "ICIQ-SF"[All Fields] OR (("urogenital system"[MeSH Terms] OR ("urogenital"[All Fields] AND "system"[All Fields]) OR "urogenital system"[All Fields] OR "urogenital"[All Fields]) AND ("distress"[All Fields] OR "distressed"[All Fields] OR "distresses"[All Fields] OR "distressful"[All Fields] OR "distressing"[All Fields]) AND ("inventoried"[All Fields] OR "inventory s"[All Fields] OR "inventorying"[All Fields] OR "personality inventory"[MeSH Terms] OR ("personality"[All Fields] AND "inventory"[All Fields]) OR "personality inventory"[All Fields] OR "inventories"[All Fields] OR "Equipment and Supplies"[MeSH Terms] OR ("equipment"[All Fields] AND "supplies"[All Fields]) OR "Equipment and Supplies"[All Fields] OR "inventory"[All Fields])) OR "UDI"[All Fields] OR ("Muscle Strength"[MeSH Terms] OR ("muscle"[All Fields] AND "strength"[All Fields]) OR "Muscle Strength"[All Fields]) OR "Muscle Strength"[MeSH Terms] OR "Muscle Strength Dynamometer"[MeSH Terms] OR (("internation"[All Fields] OR "international"[All Fields] OR "international s"[All Fields] OR "internationals"[All Fields]) AND ("consultancies"[All Fields] OR "consultancy"[All Fields] OR "consultant s"[All Fields] OR "consultants"[MeSH Terms] OR "consultants"[All Fields] OR "consultant"[All Fields] OR "consultative"[All Fields] OR "consulter"[All Fields] OR "consulters"[All Fields] OR "referral and consultation"[MeSH Terms] OR ("referral"[All Fields] AND "consultation"[All Fields]) OR "referral and consultation"[All Fields] OR "consult"[All Fields] OR "consultation"[All Fields] OR "consultations"[All Fields] OR "consulted"[All Fields] OR "consulting"[All Fields] OR "consults"[All Fields]) AND ("incontinance"[All Fields] OR "incontinence"[All Fields] OR "incontinences"[All Fields] OR "incontinency"[All Fields] OR "incontinent"[All Fields] OR "incontinents"[All Fields]) AND ("questionnair"[All Fields] OR "questionnaire s"[All Fields] OR "surveys and questionnaires"[MeSH Terms] OR ("surveys"[All Fields] AND "questionnaires"[All Fields]) OR "surveys and questionnaires"[All Fields] OR "questionnaire"[All Fields] OR "questionnaires"[All Fields])) OR (("incontinance"[All Fields] OR "incontinence"[All Fields] OR "incontinences"[All Fields] OR "incontinency"[All Fields] OR "incontinent"[All Fields] OR "incontinents"[All Fields]) AND ("impact"[All Fields] OR "impactful"[All Fields] OR "impacting"[All Fields] OR "impacts"[All Fields] OR "tooth, impacted"[MeSH Terms] OR ("tooth"[All Fields] AND "impacted"[All Fields]) OR "impacted tooth"[All Fields] OR "impacted"[All Fields]) AND ("questionnair"[All Fields] OR "questionnaire s"[All Fields] OR "surveys and questionnaires"[MeSH Terms] OR ("surveys"[All Fields] AND "questionnaires"[All Fields]) OR "surveys and questionnaires"[All Fields] OR "questionnaire"[All Fields] OR "questionnaires"[All Fields])) OR "IIQ-7"[All Fields]) AND ("Physical and Rehabilitation Medicine"[MeSH Terms] OR "Physical Therapy Modalities"[MeSH Terms] OR "Physical Therapy Modalities"[MeSH Terms] OR "Rehabilitation"[MeSH Terms] OR "Physical Education and Training"[MeSH Terms] OR "Exercise"[MeSH Terms] OR "Pelvic Floor"[MeSH Terms] OR "feedback, psychological"[MeSH Terms] OR "Equipment and Supplies"[MeSH Terms]) | | | |

Embase Research String

| Stringa: ('male'/exp OR 'male' OR 'males' OR 'man' OR 'men' OR 'stress incontinence'/exp OR 'incontinence, stress' OR 'stress incontinence' OR 'stress urinary incontinence' OR 'stress urine incontinence' OR 'urinary incontinence, stress' OR 'urinary stress incontinence' OR 'urine stress incontinence' OR 'urine incontinence'/exp OR 'bladder incontinence' OR 'incontinence, urine' OR 'incontinentia urinae' OR 'urinary incontinence' OR 'urinary leakage' OR 'urine bladder incontinence' OR 'urine incontinence' OR 'urine leakage' OR 'wetting, urine' OR 'prostatectomy'/exp OR 'prostate adenectomy' OR 'prostate resection' OR 'prostatectomy' OR 'prostatic adenectomy' OR 'radical prostatectomy' OR 'total prostatectomy') AND ('physical rehabilitation'/exp OR 'rehabilitation'/exp OR 'functional readaptation' OR 'medical rehabilitation' OR 'readaption' OR 'readjustment' OR 'rehabilitation' OR 'rehabilitation concept' OR 'rehabilitation engineering' OR 'rehabilitation potential' OR 'rehabilitation process' OR 'rehabilitation program' OR 'rehabilitation programme' OR 'rehabilitation, medical' OR 'rehabilitative treatment' OR 'resocialisation' OR 'resocialisation therapy' OR 'resocialization' OR 'resocialization therapy' OR 'revalidation' OR 'exercise'/exp OR 'biometric exercise' OR 'effort' OR 'exercise' OR 'exercise capacity' OR 'exercise performance' OR 'exercise training' OR 'exertion' OR 'fitness training' OR 'fitness workout' OR 'physical conditioning, human' OR 'physical effort' OR 'physical exercise' OR 'physical exertion' OR 'physical work-out' OR 'physical workout' OR 'pelvis floor'/exp OR 'devices'/exp OR 'apparatus' OR 'apparatus, equipment and supplies' OR 'device' OR 'devices' OR 'equipment' OR 'equipment and supplies' OR 'instrument' OR 'instrument, device' OR 'instrumentation' OR 'feedback system'/exp OR 'delayed feed back' OR 'delayed feedback' OR 'feed back control' OR 'feed back loop' OR 'feed back mechanism' OR 'feed back regulation' OR 'feed back system' OR 'feedback' OR 'feedback control' OR 'feedback control system' OR 'feedback loop' OR 'feedback mechanism' OR 'feedback regulation' OR 'feedback system' OR 'mechanism, feedback' OR 'regulation, feedback') AND 'pelvic floor muscle training'/exp AND ('urinary symptom profile'/exp OR 'muscle strength'/exp OR 'dynamic strength, muscle' OR 'dynamic strength, muscular' OR 'force, muscle' OR 'muscle dynamic strength' OR 'muscle force' OR 'muscle force velocity relationship' OR 'muscle power' OR 'muscle strength' OR 'muscular dynamic strength' OR 'muscular force' OR 'muscular power' OR 'muscular strength' OR 'strength, muscle' OR 'dynamometer'/exp OR 'biometrics m550 myometer' OR 'chatillon csd 300' OR 'cybex norm' OR 'grip-d' OR 'jamar digital pinch gauge' OR 'jamar hydraulic hand dynamometer' OR 'jamar hydraulic pinch gauge' OR 'jamar plus' OR 'jamar smedley' OR 'kin com' OR 'micro fet' OR 'multi-joint system' OR 'myogrip' OR 'myopinch' OR 'myowrist' OR 'system iii dynamometer' OR 'back/leg/chest dynamometer, electronic' OR 'dynamometer' OR 'dynamometers' OR 'electronic back/leg/chest dynamometer' OR 'jamar (dynamometer)' OR 'muscle strength dynamometer' OR 'urogenital distress inventory'/exp OR 'iciq (incontinence)'/exp OR 'iciq (incontinence)' OR 'iciq (questionnaire)' OR 'iciq (score)' OR 'iciq-sf' OR 'international consultation on incontinence questionnaire' OR 'international consultation on incontinence questionnaire short form' OR 'incontinence impact questionnaire'/exp) AND 'randomized controlled trial'/exp |
| --- |

PEDro Research String

| Abstract & title: prostatectomy  Problem: incontinence  Body part: perineum or genito-urinary system  Method: clinical trial |
| --- |

Cochrane Library Research String

| #1 MeSH descriptor: [Urinary Incontinence, Stress] 3 tree(s) exploded  #2 MeSH descriptor: [Urinary Incontinence] 3 tree(s) exploded  #3 MeSH descriptor: [Prostatectomy] explode all trees  #4 MeSH descriptor: [Male] explode all trees  #5 (#1 OR #2 OR #3) AND #4  #6 MeSH descriptor: [Physical and Rehabilitation Medicine] explode all trees  #7 MeSH descriptor: [Physical Therapy Modalities] explode all trees  #8 MeSH descriptor: [Rehabilitation] explode all trees  #9 MeSH descriptor: [Physical Education and Training] explode all trees  #10 MeSH descriptor: [Exercise] explode all trees  #11 MeSH descriptor: [Pelvic Floor] 2 tree(s) exploded  #12 MeSH descriptor: [Feedback] explode all trees  #13 MeSH descriptor: [Equipment and Supplies] explode all trees  #14 #6 OR #7 OR #8 OR #9 OR #10 OR #11 OR #12 OR #13  #15 MeSH descriptor: [Lower Urinary Tract Symptoms] explode all trees  #16 international consultation on incontinence questionnaire  #17 ICIQ-SF  #18 Urogenital Distress Inventory  #19 UDI  #20 MeSH descriptor: [Muscle Strength] explode all trees  #21 MeSH descriptor: [Muscle Strength Dynamometer] explode all trees  #22 Incontinence Impact Questionnaire  #23 IIQ-7  #24 #15 OR #16 OR #17 OR #18 OR #19 OR #20 OR #21 OR #22 OR #23  #25 #5 AND #14 AND #24 |
| --- |
