# Supplementary material for: The added value of devices to pelvic floor muscle training in radical post-prostatectomy stress urinary incontinence: A systematic review with metanalysis
Source: PLoS One. 2023 Sep 28;18(9):e0289636. doi: 10.1371/journal.pone.0289636 (PMC10538711; doi:10.1371/journal.pone.0289636)
Supplement: S2 File — (DOCX) [file pone.0289636.s002.docx]

**Supplementary Material 2**: Sensitivity analysis for the primary outcome – Electric stimulation device; (24h pad test) at week 12.
