# Supplementary material for: The added value of devices to pelvic floor muscle training in radical post-prostatectomy stress urinary incontinence: A systematic review with metanalysis
Source: PLoS One. 2023 Sep 28;18(9):e0289636. doi: 10.1371/journal.pone.0289636 (PMC10538711; doi:10.1371/journal.pone.0289636)
Supplement: Attachment — Submitted filename: Manuscript.docx [file pone.0289636.s004.docx]

**The added value of devices to pelvic floor muscle training in post-prostatectomy stress urinary incontinence: a systematic review with metanalysis**

Giardulli Benedetto^1^, Battista Simone^1^, Leuzzi Gaia^1^, Job Mirko^1^, Buccarella Ottavia^1^, Testa Marco^1^*

1. University of Genova, Department of Neurosciences, Rehabilitation, Ophthalmology, Genetics, Maternal and Child Health, Campus of Savona, Italy.

* Address all correspondence to Marco Testa at marco.testa@unige.it, Department of Neurosciences, Rehabilitation, Ophthalmology, Genetics, Maternal and Child Health, University of Genoa, Campus of Savona, Via Magliotto 2, 17100, Savona, SV, Italy.

**Running Title**: The value of devices to pelvic floor training

Giardulli Benedetto:	0000-0002-0079-0714;	benedetto.giardulli@edu.unige.it

Battista Simone:	0000-0002-7471-1951;	simone.battista@edu.unige.it

Leuzzi Gaia:	0000-0002-8478-9621;	gaialeuzzi.gl@gmail.com

Job Mirko:	0000-0002-0991-420X;	mirko.job.1991@hotmail.it

Buccarella Ottavia	0000-0001-6217-2076;	ottavia.buccarella@gmail.com

Testa Marco:	0000-0001-8643-7200;	marco.testa@unige.it

**Abstract**

**Purpose**: To investigate the role of pelvic floor devices (e.g., biofeedback, electrical stimulation, or their combination), as adjunctive treatment in pelvic floor muscle training (PFMT) in stress urinary incontinence (SUI) after prostatectomy.

**Materials and Methods**: A systematic review with meta-analysis. We searched for randomised controlled trials (RCTs) and prospective non-randomised studies investigating the effectiveness of pelvic floor devices as an adjunctive treatment for SUI symptoms assessed with weight pad-test or standardised questionnaires. To assess the risk of bias (RoB) and overall certainty of evidence, the RoB 2.0 or the ROBINS-I, and the GRADE approach were adopted.

**Results**: Eleven RCTs met our eligibility criteria. One was at a ‘low’ RoB, two had ‘some concerns’, while eight were at a ‘high’ RoB. Two meta-analyses were conducted to analyse the pooled results of five RCTs included. Specifically, two RCTs reported at week 4 with a 1h pad test a mean difference of 0.64 (95% CI= [-13.09, 14.36]), and three RCTs reported at week 12 with a 24h pad test a mean difference of -56.81 (95% CI= [-162.81, 49.19]). The heterogeneity was considerable in both the analyses (I^2^ = 80.0%; I^2^= 85.3%). The overall level of certainty was very low.

**Conclusions**: In line with our results, we cannot conclude whether pelvic floor devices add any value as adjunctive treatment in the management of SUI after prostatectomy. Future studies require more comprehensive and standardised approaches to understand whether these devices are effective.

Keywords: urology; pelvic floor disorders; urinary incontinence; rehabilitation; prostatectomy

**Introduction**

Stress urinary incontinence (SUI) is a common complication after prostatectomy (1). It is caused by both the loss of anterior and posterior anatomical supporting structures and by damages to the pelvic innervations (1,2). These injuries result in bladder or urethral sphincter dysfunctions, or both (1). Generally, 5-35% of men after prostatic surgery report urinary leakage (3), and the 95% of them describe symptoms consistent with SUI (2), even after robotic-assisted surgery (4). Furthermore, patients also experience a relevant decrease in health-related quality of life (HRQoL) after this surgery (5,6). As strongly recommended by the European Association of Urology, pelvic floor muscle training (PFMT) represents the first treatment for post-prostatectomy SUI to speed up the recovery process (7). Briefly, it consists of repetitive voluntary contractions of muscles involved in continency function such as bulbocavernosus, striated urethral sphincter and puborectalis (8,9). During the early phase of rehabilitation, different types of feedbacks are adopted to facilitate the pattern of activation of these muscles (10,11).

Feedback can be provided in different ways. First, it can be successfully provided with the supervision of a healthcare professional who verbally guides patients (12). Then, positioning a hand in perineal area can help to receive manual feedback of the contraction (13). Finally, feedback is provided using devices capable of delivering a visual or audio feedback (14,15). These devices sometimes are also used to induce a local muscle effect through electrical stimulation to improve SUI symptoms (16). However, most of these devices require an invasive anal approach that contributes to experience discomfort and to create a barrier to treatment adherence(17,18). In addition, their use is still controversial since there is no consensus on their efficacy (19,20).

In the light of the above, we performed a systematic review with meta-analysis to investigate the effect of these devices (e.g., biofeedback, electrical stimulation, or their combination) as an adjunctive treatment in the management of post-prostatectomy SUI.

**Materials and Methods**

The protocol of this systematic review was registered into the International Prospective Register of Systematic Reviews (PROSPERO; No. CRD42022307289). The reporting of this systematic review followed the Preferred Reporting Items for Systematic Reviews and Meta-Analyses statement (PRISMA) 2020 (21). To conduct this systematic review the Cochrane Handbook for Systematic Reviews of Interventions was used (22).

*Objective*

The primary aim of this systematic review is to analyse the effect of pelvic floor devices (electrostimulation, biofeedback etc.) as adjunctive treatments in a PFMT programme in post-prostatectomy SUI.

*Eligibility criteria*

*Type of Publications*

Only randomised control trials (RCTs) and prospective non-randomised studies written in English were taken into account. Case series, single-case studies were excluded from the analysis. No limitations on the publication date were set. Abstracts and reports from meetings were excluded.

*Population*

For this systematic review, only studies addressing male adults (age > 18 years) with post-prostatectomy SUI were considered eligible. No follow-up, symptoms duration and symptom severity limits were set. On the other hand, any type of comorbidity that could interfere with the pelvic floor training results (e.g., chronic, or acute neurologic diseases; ongoing prostate cancer ; surgery neurologic injuries) were reasons for exclusion. Patients with a history of cancer other than recent prostatectomy were not excluded. Any other UI types, such as urge incontinence were also excluded.

*Types of Intervention*

Studies that investigated the use of a pelvic floor device (e.g. biofeedback or electrostimulation) as an adjunctive therapy in the management of post-prostatectomy SUI were considered eligible. Therefore, studies needed to compare the effectiveness of PFMT with and without the combined use of a device. Any permanent implantable or surgical device was not considered eligible. PFMT was considered as any training involving specifically the contraction of pelvic floor muscles, both supervised and not. No limits on duration or frequency were set. Studies that evaluated a pelvic floor device in isolation were excluded.

*Types of Outcomes*

The primary outcome of this study was the pad weight test, since it is considered a gold standard to quantitatively assess urinary losses or, alternatively, any other gold standard and self-reported tool to assess UI symptoms (e.g. international consultation on incontinence questionnaire; ICIQ). No limits on repetitions (e.g. 1h, 4h etc.) were set. The secondary outcome was HRQoL.

*Search strategy*

As suggested from the Cochrane Handbook for Systematic Reviews of Interventions(22) we chose Pubmed, EMBASE and Cochrane Library-CENTRAL and a specific electronic database based on the research question. Therefore, we also performed our research on PEDro. We systematically performed the research on these databases up to 27 January 2022.

The search was conducted by three authors (B.G., S.B., and G.L.). The search strategy was a combination of Medical Subjects Headings, Boolean operators (e.g. AND and OR) and the keywords “urinary incontinence”, “stress”, “prostatectomy”, “male”, “physical and rehabilitation medicine”, “pelvic floor”, “exercise”, “feedback”, “lower urinary tract symptoms”, and “incontinence impact questionnaire”. The research strings for every database are reported in the Supplementary Materials.

*Selection process*

Articles were uploaded onto Rayyan Website after duplicate removal (23). Afterwards, two researchers (B.G. and G.L.) independently and systematically carried out the starting search applying the inclusion and exclusion criteria to titles and abstracts. When the authors were in disagreements a third author (S.B.) was consulted to reach a consensus. No author or expert were contacted to get additional studies. When necessary, the full text was read.

*Data collection*

Two researchers (B.G. and G.L.) independently extracted the following data from each study using standardised Excel templates: authors, year of publication, country, setting, study design, the total number of participants, age, number in each group, type of intervention and control, the timing of administration of intervention and baseline, post-intervention and follow-up (when available) points estimates, measures of variability of main outcomes and authors key conclusion. Results for both primary and secondary outcomes were extracted. To be able to make a comparison between outcomes and to facilitate the eventual meta-analysis, data were divided based on the times of assessment (e.g. 2 weeks, 4 weeks) and the tests adopted (e.g. 1h pad test, 4h pad test). Authors of studies where data were not completely displayed were contacted. In case of disagreement in the data extraction process, a third author (S.B.) was consulted to gain a consensus.

*Study risk of bias*

The risk of bias and methodological quality of the included studies were independently assessed by two authors (B.G. and G.L.). For randomised controlled trials we used the Revised Cochrane risk-of-bias tool 2.0 (RoB 2.0), recommended to assess risk of bias in Cochrane Reviews (24) . This tool allows for assessing on a standard set of items used for the risk of bias appraisal: “bias arising from the randomisation process”, “bias due to deviations from intended interventions”, “bias due to missing outcome data”, “bias in the measurement of the outcome”, “bias in the selection of the reported result”, and, finally, the risk of bias judgment for each outcome. For prospective studies, instead, we used the Risk of Bias in a non-randomised study (ROBINS-I) (25). This tool allows for assessing on a standard set of items used for the risk of bias appraisal: “bias due to confounding”, “bias in selection of participants into the study”, “bias in classification of interventions”, “bias due to deviations from intended interventions”, “bias due to missing data”, “bias in measurement of outcomes” and “bias in selection of the reported result”. Both the tools, recommended by the Cochrane Library, allowed the studies to be classified as “low”, “some concerns”, or “high” risk of bias. In case of disagreement between the reviewers, a consensus was obtained after the consultation of a third one (S.B.).

*Data analysis and synthesis*

Statistical analysis was done via Review Manager 5.3 (RevMan-Copenhagen: The Nordic Cochrane Center, The Cochrane Collaboration, 2014) and Stata 17 (StataCorp). For inter-groups comparisons, the mean, the standard deviation, and/or mean differences and the 95% CI were extracted when available or calculated when possible. Medians and interquartile ranges were extracted, when mean, the standard deviation, and/or mean differences were not presented. Continuous data were combined through meta-analysis using a random-effect model when appropriate. As we expected different measures to assess the primary outcome (pad test), the standardised mean difference (SMD) was adopted. Statistical heterogeneity was assessed using the I^2^ statistic. The overall certainty of the evidence and strength of the recommendations were evaluated using the Grading of Recommendations Assessment, Development and Evaluation (GRADE) approach (26) through the GRADEpro GDT (https://gradepro.org, accessed date: 16 July 2022), for the primary outcome (pad test). The downgrading process was based on five domains: study limitations (e.g., risk of bias), inconsistency (e.g., heterogeneity between study results), indirectness of evidence (generalisability and transferability, e.g., short-term follow-up), imprecision (e.g., small sample size), and reporting bias (e.g., publication bias).

**Results**

*Study selection*

Database searches initially yielded 4526 articles. After removing the duplicates, they were reduced to 4240. Of these, after the screening selection through titles and abstracts, 22 were screened for eligibility. Finally, after the full-text screening, only 11 met the inclusion criteria and were considered for our critical review (27–37). Figure 1 reports the flow diagram that thoroughly reports the study selection process^31,32,34,36,37^. The studies were published between 1999 and 2021. Of the 11 studies included in the review, all studies were prospective RCTs that had at least two treatments (one with and one without pelvic floor devices). Only one prospective study reached the last screening phase, but it was excluded because there was only one treatment without a control. Sample size of post-prostatectomy SUI ranged from 13 to 332 across the studies. The pooled population comprised 1156 participants. Follow-up during treatment ranged from 1 to 3 months.

*Study characteristic*

Table 1 reports the main characteristics of each study. Three studies were conducted in Brazil (27,29,35), one in Egypt (37), one in China (36), one in Italy (33), one in Canada(32), one in South Korea (31), one in Poland (30) and one in Germany(28). The country of one study was unknown (34). We contacted the authors to retrieve this information but we did not receive any answer. As far as the SUI symptoms are concerned, four studies investigated them with a 24h pad test (29–32,37), four studies with a 1h pad test(33–36), one with the 20min pad test (28), and one with the ICIQ (33). Only one study used both the 1h pad test and the 24h pad test (30). HRQoL was assessed in eight studies out of twelve (27,29–33,36,37), with different scales. Three used the IIQ-7 (29,30,37), two used the ICIQ-SF (27,36), one used both the IIQ-7 and the EORTC QLQ C30 (32), one used the IPSS-QoL (31), and one used RAND 36 – Item Health Survey (33).

Table 1. Characteristics of the studies included.

| **Authors** | **Title** | **Year** | **N°** | **Device used** | **Intervention** | **Control** | **Outcome** | **Follow-up** | **Treatment time and n° of sessions** |
| --- | --- | --- | --- | --- | --- | --- | --- | --- | --- |
| Ahmed M.T., Mohammed A.H. and Amansour A. | Effect of Pelvic Floor Electrical Stimulation and Biofeedback on the Recovery of Urinary Continence after Radical Prostatectomy | 2012 | 80 | a 2-channel electromyographic BFB apparatus was used with one channel for perineal, and the other for abdominal muscles | Group 1: P PFMT + Electric Stimulation  Group 2: PFMT + Electric Stimulation + Biofeedback | PFMT not supervised | 24 h pad test  IIQ-7 | W0  W6  W12  W24 | 15 min  Twice weekly for 12 weeks |
| An D., Wang J., Zhang F., Wu J., Jing H., Gao Y., Cong H., et al. | Effects of Biofeedback Combined With Pilates Training on Postprostatectomy Incontinence | 2021 | 42 | Rectal probe surface electrode | Group B: PFMT + Biofeedback  Group C: PFMT + Biofeedback + Pilates | Group A: PFMT not supervised | 1 h pad test  ICIQ-SF  IEF  Oxford Grading Scale | W0  W4  W8 | 40 min  Daily for 8 weeks |
| De Santana N.A., De Lima Saintrain M.V., Regadas R.P. et al. | Assessment of Physical Therapy Strategies for Recovery of Urinary Incontinence after Prostatectomy | 2017 | 13 | Anal manometry inflated with 15 ml of air | PFMT + Biofeedback | PFMT supervised | 1 h pad test | W0  W4  W8 | 20 min  Once a week for 8 weeks |
| Floratos D.L., Sonke G.S., Rapidou C.A., Alivizatos G.J., et al. | Biofeedback vs Verbal biofeedback as learning tools for pelvic muscle exercises in the early management of urinary incontinence after prostatectomy | 2002 | 42 | a 2-channel electromyographic BFB apparatus was used with one channel for perineal, and the other for abdominal muscles | PFMT + Biofeedback | PFMT supervised | 1 h pad test | M1 M2 M3  M6 | 30 min  Three a week for 5 weeks |
| Laurienzo C.E., Magnabosco W.J., Jabur F., Faria E.F., Gameiro M.O., et al. | Pelvic floor muscle training and electrical stimulation as rehabilitation after radical prostatectomy, a randomized controlled trial | 2018 | 123 | Anal electro-stimulator | Group 1: Routine instructions  Group 3: PFMT + Electro-stimulation | Group 2: PFMT not supervised | 1 h pad test  Perinometers  ICIQ-SF  IIEF-5  IPSS | M1  M3  M6 | Not specified  Twice a week for 7 weeks |
| Marchiori D., Bertaccini A., Manferrari F, Ferri C. and Martorana G. | Pelvic Floor Rehabilitation for Continence Recovery after Radical Prostatectomy: Role of a Personal Training Re-Educational Program | 2010 | 332 | Not specified | PFMT + Biofeedback + Electrical Stimulation | PFMT not supervised | ICIQ  RAND 36-Item Health Survey | M3 M6 M12 | 15 min  Mean period of 2-3 weeks |
| Moore K.N., Griffiths D. and Hughton A. | Urinary incontinence after radical prostatectomy: a randomized controlled trial comparing pelvic muscle exercises with or without electrical stimulation | 1999 | 58 | Surface anal electrode | Group 2: Intensive PFMT  Group 3: PFMT + Electrical stimulation | Group 1: PFMT not supervised | 24 h pad test  IIQ-7  EORTC QLQ C30  General Urology Symptom Inventory | W0  W12  W16  W24 | 30 min  Twice a week for 12 weeks |
| Oh J.J., Kim J.K., Lee H., Lee S., Jeong S.J., Hong S.K., Lee S.E. and Byun S. | Effect of personalized extracorporeal biofeedback device for pelvic floor muscle training on urinary incontinence after robot-assisted radical prostatectomy: a randomized controlled trial | 2019 | 82 | Portable extracorporeal perineometer | PFMT + Biofeedback | PFMT not supervised | 24 h pad test  IPSS  IIEF-5 | M0  M1  M2  M3 | 10 min  4 times per day |
| Rajkowska-labon E., Bakula S., Kucharzexski M and Sliwinski Z. | Efficacy of physiotherapy for urinary incontinence following prostate cancer surgery | 2014 | 81 | Anal probe with electromyography | Group II: no therapy  Group IA: PFMT + biofeedback + spinal segmental stabilisation | Group IB: PFMT + spinal segmental stabilisation | 1 h pad test  24 h pad test  sEMG muscle tension | Y0  Y1 | 30 min  Twice weekly |
| Riberio L.H.S., Prota C., Gomes C.M., de Bessa J., Boldarine M.P., Dall’Oglio M.F., Bruschini H. and Srougi M. | Long-term effect of early postoperative pelvic floor biofeedback on continence in men undergoing radical prostatecomy: a prospective, randomized, controlled trial | 2010 | 73 | Surface anal electrode | PFMT + biofeedback | PFMT not supervised | ICSI  ICST  IIQ-7  Oxford scale | M1  M3  M6  M12 | 30 min  Once a week until continent or for a maximum of 12 weeks |
| Willie S., Sobottka A., Heidenreich A. and Hofmann R. | Pelvic floor exercises, electrical stimulation and biofeedback after radical prostatectomy: results of a prospective randomized trial | 2003 | 139 | Bioimpulser surface anal electrode. | Group 2: PFMT + Electric stimulation  Group 3: PFMT + Electric stimulation + biofeedback | Group 1: PFMT not supervised | 20-minute pad test | M0  M3  M12 | 15 min ES  15 min BFB  Twice daily for 3 months |
| Legend: N, numer; Int., intervention; Cont., control; W, week; M, month; Y, year; PFMT, pelvic floor muscle training; sEMG, electromyographic signal; ES, electric stimulation; BFB, biofeedback; h, hour; IIQ-7, incontinence impact questionnaire short form; ICIQ-SF, international consultation on incontinence questionnaire short form; IEF, episodes of incontinence; IIEF-5, international index of erectile function; IPSS, international prostatic symptoms score; ICIQ, international consultation on incontinence questionnaire; EORTC QLQ C30, european organisation for research and treatment of cancer core quality of life questionnaire; ICSI, incontinence symptoms of the international continence society male short form; ICST, total score of the international continence society male short form questionnaire. | | | | | | | | | |

*Risk of bias in studies*

The risk of bias assessment of RCTs is displayed in Figure 2. Among the included studies, one was at a “low risk”, two had “some concerns”, while eight were at a “high risk”. In general, data were not available for all the participants included in the studies and there were no analysis methods that correct for bias or sensitivity analysis. Moreover, it was not always possible to have a pre-specified analysis protocol (28–30,32–37). We contacted all authors to receive further information or raw data but only two of them replied (33,36).

*Results of individuals studies*

Available results of individual studies are presented in Table 2 and Table 3. Among the studies, there were different treatment arms: eight arms were PFMT not supervised (27–29,31–33,36,37); four were PFMT supervised (30,32,34,35); two were no therapy (30,34); six were PFMT + BFB (29–31,34–36); four were PFMT + ES (28,32,33,37); two were PFMT + BFB + ES (28,37); finally, one was PFP + Pilates (36). Arms with PFMT supervised and not were considered as control for this review. The treatment methods in terms of frequency and time were different among studies. Sessions lasted from a minimum of 5 minutes to a maximum of 40 minutes.

Table 2. Primary outcome (pad test) of the studies included.

| **Author, year** | **Outcome measure** | **Groups** | **Baseline** | **T1** | **T2** | **T3** |
| --- | --- | --- | --- | --- | --- | --- |
| **Pad weight test and UI symptoms (Primary outcome)** | | | | | | |
| Ahmed  2012 | 24h pad test | PFMT + ES | 790 ± 399.46 | 383 ± 145.87 | 132 ± 145.87 | 97.8 ± 105.87 |
|  |  | PFMT + ES + BFB | 785 ± 311.98 | 263 ± 145.87 | 83 ± 145.87 | 36 ± 95.87 |
|  |  | PFMT NS | 791 ± 380.3 | 533 ± 316.53 | 260 ± 216.53 | 123 ± 116.53 |
| An  2021 | 1h pad test | PFMT + BFB | 58.64 ± 8.72 | 45.93 ± 7.63 | 22.29 ± 4.82 |  |
|  |  | PFMT + Pilates | 56.51 ± 9.46 | 41.43 ± 5.94 | 18.29 ± 2.4 |  |
|  |  | PFMT NS | 57.01 ± 8.46 | 51.46 ± 7.55 | 37.43 ± 7.36 |  |
| De Santana  2017 | 1h pad test | PFMT + BFB | No data |  |  |  |
|  |  | PFMT S | No data |  |  |  |
| Floratos  2002 | 1h pad test | PFMT + BFB | 42 ± 33.33 | 20.52 ± 24.63 | 9.08 ± 12.55 | 7.06 ± 11.7 |
|  |  | PFMT S | 34 ± 27.44 | 12 ± 11.63 | 5.06 ± 6.49 | 3.7 ± 4.25 |
| Laurienzo  2018 | 1h pad test | No intervention | 1 (0-22) | 5 (3-351) | 1 (0-279) | 1 (0-231) |
|  |  | PFMT + ES | 0.5 (0-36) | 9 (3-241) | 1 (0-183) | 1 (0-18) |
|  |  | PFMT NS | 1 (0-3) | 7 (3-431) | 2 (0-74) | 1 (0-78) |
| Marchiori  2010 | ICIQ-Male | PFMT + ES | No data |  |  |  |
|  |  | PFMT NS | No data |  |  |  |
| Moore  1999 | 24h pad test | PFMT S | 565.6 ± 403.3 | 86.9 ± 123 | 73.5 ± 131.4 | 69.9 ± 113.5 |
|  |  | PFMT + ES | 452.5 ± 385.1 | 155.5 ± 168.1 | 202.2 ± 242.2 | 98.2 ± 131.1 |
|  |  | PFMT NS | 385.9 ± 256.9 | 103.8 ± 176.3 | 67.3 ± 137.4 | 54.1 ± 103.1 |
| Oh  2019 | 24h pad test | PFMT + BFB | No data | 71 ± 48 | 59.7 ± 83.4 | 38.8 ± 141.2 |
|  |  | PFMT NS | No data | 120.8 ± 132.7 | 53.1 ± 96.6 | 19.5 ± 57.2 |
| Rajkowska-labon  2014 | 1h pad test | No intervention | No data |  |  |  |
|  |  | PFMT S | No data |  |  |  |
|  |  | PFMT + BFB | No data |  |  |  |
|  | 24h pad test | No intervention | 61.6 (32.04-12.6) | 12.71 (4.14-17.13) | No data |  |
|  |  | PFMT S |  |  |  |  |
|  |  | PFMT + BFB | No data |  |  |  |
| Riberio  2010 | 24h pad test | PFMT + BFB | 28 (8-82) | 6 (0-24) | 2 (0-12.5) | 0 (0-3) |
|  |  | PFMT NS | 49 (15-605) | 58 (18-210) | 8 (0-164) | 4 (0-70) |
| Willie  2003 | 20m pad test | PFMT + ES | 35.22 | 75.4 | 80.34 |  |
|  |  | PFMT + ES + BFB | 34.3 | 72.28 | 89.56 |  |
|  |  | PFMT NS | 29.92 | 63.54 | 75.5 |  |
| Legend: UI, urinary incontinence; h, hour; m, minute; T, time; PFMT, pelvic floor muscle training; ES, electric stimulation; BFB, biofeedback; NS, not supervised; S, supervised. | | | | | | |

Table 3. Secondary outcome (health-related quality of life) of the studies included.

| **Author, year** | **Outcome measure** | **Groups** | **Baseline** | **T1** | **T2** | **T3** |
| --- | --- | --- | --- | --- | --- | --- |
| **HRQoL (Secondary outcome)** | | | | | | |
| Ahmed  2012 | IIQ-7 | PFMT + ES | 54 ± 26 | 36 ± 25 | 29 ± 28 | 23 ± 24 |
|  |  | PFMT + ES + BFB | 53 ± 28 | 26 ± 25 | 20 ± 24 | 15 ± 25 |
|  |  | PFMT NS | 55 ± 31 | 40 ± 23 | 32 ± 26 | 25 ± 26 |
| An  2021 | ICIQ-SF | PFMT + BFB | 16 (16-18) | 12 (10-13) | 8 (7-9) |  |
|  |  | PFMT + Pilates | 17 (16-19) | 10 (11-12) | 6 (5-8) |  |
|  |  | PFMT NS | 17 (16-18) | 15 (13.15) | 12 (11-14) |  |
| Laurienzo  2018 | ICIQ-SF | No intervention | 0 (0-18) | 8 (1-21) | 6 (0-21) | 4 (0-21) |
|  |  | PFMT + ES | 0 (0-18) | 11 (1-21) | 5.5 (0-20) | 4 (0-18) |
|  |  | PFMT NS | 0 (0-14) | 11 (1-21) | 6 (0-17) | 3 (0-16) |
| Marchiori  2010 | RAND 36-Item Health Survey | PFMT + ES | No data |  |  |  |
|  |  | PFMT NS | No data |  |  |  |
| Moore  1999 | IIQ-7 | PFMT S | No data |  |  |  |
|  |  | PFMT + ES | No data |  |  |  |
|  |  | PFMT NS | No data |  |  |  |
|  | EORTC QLQ C30 | PFMT S | No data |  |  |  |
|  |  | PFMT + ES | No data |  |  |  |
|  |  | PFMT NS | No data |  |  |  |
| Oh  2019 | IPSS-QOL | PFMT + BFB | 2.8 ± 1.6 | -1.13 ± 1.65 | -0.9 ± 1.5 | -0.33 ± 1.39 |
|  |  | PFMT NS | 3.1 ± 1.3 | -0.93 ± 2.02 | -0.57 ± 2.06 | 0.05 ± 2.06 |
| Rajkowska-labon  2014 | IIQ-7 | No intervention | No data |  |  |  |
|  |  | PFMT S | No data |  |  |  |
|  |  | PFMT + BFB | No data |  |  |  |
| Riberio  2010 | IIQ-7 | PFMT + BFB | 3* | 2.4 | 0.5 | 0.7 |
|  |  | PFMT NS | 7.2* | 4 | 2.8 | 1.6 |
| Legend: HRQoL, health-related quality of life; T, time; PFMT, pelvic floor muscle training; ES, electric stimulation; BFB, biofeedback; NS, not supervised; S, supervised; IIQ-7, incontinence impact questionnaire short form; ICIQ-SF, international consultation on incontinence questionnaire short form; IPSS-QOL, international prostatic symptoms score quality of life; EORTC QLQ C30, european organisation for research and treatment of cancer core quality of life questionnaire; *, change in mean. | | | | | | |

*Primary outcome – weight pad test*

Regarding the weight pad test, five studies found that the intervention group reduced this outcome compared to the control group (29,30,35–37). Two studies, on the other hand, as in the previous studies, found a reduction compared to the control group, but their magnitude was lower (31,33). Finally, four studies found no difference between the intervention and control groups (27,28,32,34).

*Secondary outcome – HRQoL*

As far as HRQoL, three studies reported that there was no difference between the intervention and control groups (27,29,31). Only two studies reported an increase in HRQoL in the intervention group compared to the control group (36,37). Three studies did not provide any raw data concerning this outcome (30,32,33). We have contacted the authors to collected them, but we received no answer.

*Results of synthesis*

Based on the availability of the outcome times of assessment and the test used, between the studies, five studies were eligible for meta-analysis (31,32,34,36,37). Authors were contacted to provide the missing data to possibly extend the meta-analysis. Two different meta-analyses were conducted based on the availability of the primary outcome data 24h and 1h pad test at 4 and twelve weeks, respectively. Pooled results of the use of a device in addition to PFMT in urinary loss, assessed with weight pad test, reported at week 4 with a 1h pad test a mean difference of 0.64 (CI= -13.09, 14.36), and at week 12 with a 24h pad test a mean difference of -56.81 (CI= -162.81, 49.19). The heterogeneity was I^2^ = 80.0%; I^2^= 85.3% respectively. Figure 3 and Figure 4 summarise the results of meta-analyses.

*Reporting biases*

It was not possible to investigate the bias of publication for the meta-analyses due to the low numbers of studies (<10 studies), as reported in the Cochrane Handbook for Systematic Reviews of Interventions (22).

*Certainty of evidence*

In Table 4 are reported the GRADE assessments. The overall certainty of the evidence was very low.

Table 4. GRADE approach assessment.

| Certainty assessment | | | | | | | N° of patients | | Effect | |  |
| --- | --- | --- | --- | --- | --- | --- | --- | --- | --- | --- | --- |
| N° of Studies | Study Design | Risk of Bias | Inconsistency | Indirectness | Imprecision | Other Considerations | PFMT + device | PFMT alone | Relative (95% CI) | Absolute (95% CI) | Certainty |
| Urine loss (Follow-Up: twelve weeks; Assessed with: 24h Pad test) | | | | | | | | | | | |
| 3 | RCT | Very serious ^a^ | Very serious ^b^ | Not serious | Serious ^c^ | None | 121 | 123 | // | -56.81 (-162.81, 49.19) | ⊕◯◯◯  Very low^†^ |
| Urine loss (Follow-Up: four weeks; Assessed with: 1h Pad test) | | | | | | | | | | | |
| 2 | RCT | Serious ^d^ | Very serious ^b^ | Not serious | Serious ^c^ | None | 42 | 28 | // | 0.64 (-13.09, 14.36) | ⊕◯◯◯  Very low^†^ |

**Discussion**

This systematic review with meta-analysis is the first one that tested the efficacy of devices as adjunctive treatment to PFMT in the management of post-prostatectomy SUI.

Among the eleven studies included in the review, seven supported the use of a device in addition to PFMT alone (29–31,33,35–37). On the other hand, four studies reported no difference between the group with a device and the one with PFMT alone (27,28,32,34). From the pooled results of the two meta-analyses and the GRADE assessment, we found a high heterogeneity among studies with a level of evidence very uncertain. In line with that, we could not conclude whether the adjunctive use of devices may enhance or not improve SUI symptoms following prostatectomy. This contrasts with the results of a review by Sciarra et al. that summarised the evidence upon the biofeedback and electric stimulation for post-prostatectomy UI. In their review, they affirmed that devices in the management of UI following prostatectomy improved incontinence recovery rate within the first 3 months compared with PFMT alone (38). However, their review was not focused on SUI symptoms, and did not provide a level of certainty of the reported results (e.g., GRADE approach).

The huge variability of results is in line with previous evidence, also about the role of PFMT alone. Cochrane reported that PFMT has conflicting evidence at twelve months post-surgery, concluding that the role of conservative treatment remains uncertain(20). Conversely, there are different studies that suggest the crucial role of PFMT in recovering or speeding-up SUI symptoms (12,39–41). The coexistence of these results may be linked to the different ways in which PFMT is delivered, contributing to the high heterogeneity of PFMT treatment outcomes.

The first element to consider is whether the PFMT is supervised by a healthcare professional or not, since it has been observed that it may contribute to reach better outcome, as shown by Wu M. and colleagues (12). Among the studies included, there were controls groups that received only routine exercises (provided with a pamphlet) compared to interventions with supervised PFMT plus the use of a device. Only four had a control with a supervised PFMT (30,32,34,35).This kind of imbalance between intervention and control group might have biased the analysis of the results favouring the intervention groups. However, this is just guesswork as a few studies reported that routine instructions brought similar levels of improvement compared to supervised PFMT (42,43).

Secondly, the PFMT load, expressed in time and frequency, might affect the SUI symptoms outcome (44). Currently there are no standardised protocols to take as a reference (45), therefore studies perform different loads. García-Sánchez and colleagues, in their review reported that, independently from the load adopted, all women with SUI improved their symptoms, however they reported that larger effects were reached with a load lasting 6–12 weeks, with >3 sessions/week and a length of session <45 min (46). The loads adopted by the studies included in our review were all different from each other, and only three of them were in line with the load suggested by García-Sánchez et al on women (28,31,36), but their results were different and not consistent with one another.

Finally, the intervention itself, based on the provided exercises, may contribute to reach different outcomes. Hodges P. et al. reported that to recruit the muscles involved in urinary continence in men better, a focus of anterior pelvic muscles is essential (13). Therefore, the most relevant command to provide to patients is “shorten the penis” (47). Moreover, Kruger et al. reported that PFMT requires specifically the muscle recruitment of pelvic floor muscles, and not accessory muscles (e.g. hips, gluteus, abdominals), otherwise the contraction will not be sufficient to bring to an effect (48,49). Nevertheless, none of the studies included in our review had a focus on anterior pelvic muscles. Ten studies reported to provide a biofeedback or electrical stimulation of anal muscles (27–30,32–37). One study adopted control exercises focused on hips adductors and abdominals (27). Lastly, one study did not mention exercises on which muscles were focused (31).

Given the overall controversial results from this review, we wonder whether, in clinical practice, it is helpful to adopt devices with an intrusive anal approach in a population who already experience urinary incontinence as a taboo (5,6). People with SUI do not consult health professionals for management and treatment due to its negative impact on their privacy and sexuality (50). The added discomfort experienced from these devices may result in a ‘barrier’ to a treatment whose efficacy is still controversial. Future studies might also give voice to patients and explore these aspects to understand the perceived usefulness of these treatments.

From the GRADE approach, this review found a very low certainty of evidence. More studies with a strong and accurate design are needed in the scientific literature to shed some light on the real efficacy of devices in the PFMT management of post-prostatectomy stress UI. Future studies need to reduce the gaps between the control and intervention arms, to implement a better blinding process, and to define in detail the timing, frequency, and delivery modalities of treatments to ease the comparing process.

Some limits must be reported. First, the high heterogeneity among studies in terms of pad weight (I^2^>80%), which might be linked to surgical prostatectomy procedures that were not assessed in this study. Secondly, not all studies included reported the characteristics of the population (e.g., age, pad weight) and sometimes the structure of the studies was inconsistent. Studies differed in the reported outcomes and time assessments and in the different interventions delivered, both in modality and load exercise. Finally, we could not assess publication bias due to the low number of studies included in the analysis.

**Conclusions**

This review found a very low level of certainty in the evidence and a high level of heterogeneity. Therefore, we cannot conclude whether pelvic floor devices are useful as adjunctive treatment in SUI after prostatectomy. Future studies require more comprehensive and standardised approaches to understand whether these devices are effective as adjunctive treatment in this disease.

**Author Contributions**: all authors contributed to the study conception and design. Benedetto Giardulli, Simone Battista, Marco Testa did conceptualisation. Simone Battista performed methodology; Benedetto Giardulli, Gaia Leuzzi did formal analysis and investigation; Benedetto Giardulli, Mirko Job, Ottavia Buccarella did writing – original draft preparation; Benedetto Giardulli, Mirko Job, Ottavia Buccarella did writing – review and editing; Benedetto Giardulli and Simone Battista performed statistical analysis. All Authors read and approved the final manuscript.

**Funding**: nil.

**Conflicts of Interest**: the authors declare no conflicts to disclose.

**Ethical Committee Approval**: nil.

**Author Responsibilities**: nil.

**Acknowledgments**: nil.

**References:**

1.	Heesakkers J, Farag F, Bauer RM, Sandhu J, de Ridder D, Stenzl A. Pathophysiology and Contributing Factors in Postprostatectomy Incontinence: A Review. Eur Urol [Internet]. 2017 Jun 1;71(6):936–44. 2.	Singla N, Singla AK. Post-prostatectomy incontinence: Etiology, evaluation, and management. Turk J Urol [Internet]. 2014 Mar;40(1):1. 3.	Ficarra V, Novara G, Rosen RC, Artibani W, Carroll PR, Costello A, et al. Systematic Review and Meta-analysis of Studies Reporting Urinary Continence Recovery After Robot-assisted Radical Prostatectomy. Eur Urol. 2012 Sep 1;62(3):405–17.

4.	Averbeck MA, Woodhouse C, Comiter C, Bruschini H, Hanus T, Herschorn S, et al. Surgical treatment of post-prostatectomy stress urinary incontinence in adult men: Report from the 6th International Consultation on Incontinence. Neurourol Urodyn [Internet]. 2019 Jan 1 ;38(1):398–406.

5.	Corrado B, Giardulli B, Polito F, Aprea S, Lanzano M, Dodaro C. The Impact of Urinary Incontinence on Quality of Life: A Cross-Sectional Study in the Metropolitan City of Naples. Geriatrics (Basel) [Internet]. 2020 Dec 1 [cited 2022 Aug 7];5(4):1–14.

6.	Bernardes MFVG, Chagas S de C, Izidoro LC de R, Veloso DFM, Chianca TCM, da Mata LRFP. Impact of urinary incontinence on the quality of life of individuals undergoing radical prostatectomy. Rev Lat Am Enfermagem [Internet]. 2019 [cited 2022 Aug 7];27.

7.	Burkhard FC, Bosch JLHR, Cruz F, Lemack GE, Nambiar AK, Thiruchelvam N, et al. EAU GUIDELINES ON URINARY INCONTINENCE.

8.	Nahon I. Physiotherapy management of incontinence in men. J Physiother [Internet]. 2021 Apr 1 [cited 2022 Aug 4];67(2):87–94. 9.	Stafford RE, Ashton-Miller JA, Constantinou C, Coughlin G, Lutton NJ, Hodges PW. Pattern of activation of pelvic floor muscles in men differs with verbal instructions. Neurourol Urodyn [Internet]. 2016 Apr 1 [cited 2022 Aug 4];35(4):457–63.

10.	Magill RA, Anderson DI. Motor learning and control : concepts and applications. Eleventh edition. New York  NY: McGraw-Hill Education; 2017. 781 p.

11.	Schmidt R, Lee T. Motor Control And Learning: A Behavioral Emphasis, Fourth Edition. 2005 [cited 2022 Aug 4];544.

12.	Wu MLY, Wang CS, Xiao Q, Peng CH, Zeng TY. The therapeutic effect of pelvic floor muscle exercise on urinary incontinence after radical prostatectomy: a meta-analysis. Asian J Androl [Internet]. 2019 Mar 1 [cited 2022 Aug 4];21(2):170.

13.	Hodges PW, Stafford RE, Hall L, Neumann P, Morrison S, Frawley H, et al. Reconsideration of pelvic floor muscle training to prevent and treat incontinence after radical prostatectomy. Urol Oncol [Internet]. 2020 May 1 [cited 2022 Aug 4];38(5):354–71.

14.	Lee HN, Lee SY, Lee YS, Han JY, Choo MS, Lee KS. Pelvic floor muscle training using an extracorporeal biofeedback device for female stress urinary incontinence. Int Urogynecol J [Internet]. 2013 May [cited 2022 Aug 4];24(5):831–8.

15.	Scott KM. Pelvic Floor Rehabilitation in the Treatment of Fecal Incontinence. Clin Colon Rectal Surg [Internet]. 2014 [cited 2022 Aug 4];27(3):99. Available from: /pmc/articles/PMC4174224/

16.	Li W, Hu Q, Zhang Z, Shen F, Xie Z. Effect of different electrical stimulation protocols for pelvic floor rehabilitation of postpartum women with extremely weak muscle strength: Randomized control trial. Medicine [Internet]. 2020 Apr 1 [cited 2022 Aug 4];99(17):e19863. Available from: /pmc/articles/PMC7440138/

17.	Hay-Smith J, Dean S, Burgio K, McClurg D, Frawley H, Dumoulin C. Pelvic-floor-muscle-training adherence “modifiers”: A review of primary qualitative studies-2011 ICS State-of-the-Science Seminar research paper III of IV. Neurourol Urodyn [Internet]. 2015 Sep 1 [cited 2022 Aug 4];34(7):622–31. Available from: https://pubmed.ncbi.nlm.nih.gov/25998067/

18.	Elenskaia K, Haidvogel K, Heidinger C, Doerfler D, Umek W, Hanzal E. The greatest taboo: urinary incontinence as a source of shame and embarrassment. Wien Klin Wochenschr [Internet]. 2011 Oct [cited 2022 Aug 4];123(19–20):607–10. Available from: https://pubmed.ncbi.nlm.nih.gov/21935649/

19.	Goode PS, Burgio KL, Johnson TM, Clay OJ, Roth DL, Markland AD, et al. Behavioral therapy with or without biofeedback and pelvic floor electrical stimulation for persistent postprostatectomy incontinence: a randomized controlled trial. JAMA [Internet]. 2011 Jan 12 [cited 2022 Aug 4];305(2):151–9. Available from: https://pubmed.ncbi.nlm.nih.gov/21224456/

20.	Anderson CA, Omar MI, Campbell SE, Hunter KF, Cody JD, Glazener CMA. Conservative management for postprostatectomy urinary incontinence. Cochrane Database Syst Rev [Internet]. 2015 Jan 20 [cited 2022 Aug 4];1(1). Available from: https://pubmed.ncbi.nlm.nih.gov/25602133/

21.	Page MJ, McKenzie JE, Bossuyt PM, Boutron I, Hoffmann TC, Mulrow CD, et al. The PRISMA 2020 statement: an updated guideline for reporting systematic reviews. BMJ [Internet]. 2021 Mar 29 [cited 2022 Aug 3];372. Available from: https://www.bmj.com/content/372/bmj.n71

22.	Higgins JPT, Thomas J, Chandler J, Cumpston M, Li T, Page MJ, et al. Cochrane Handbook for Systematic Reviews of Interventions version 6.3 (updated February 2022). Cochrane. 2022.

23.	Ouzzani M, Hammady H, Fedorowicz Z, Elmagarmid A. Rayyan-a web and mobile app for systematic reviews. Syst Rev [Internet]. 2016 Dec 5 [cited 2022 Aug 3];5(1):1–10. Available from: https://systematicreviewsjournal.biomedcentral.com/articles/10.1186/s13643-016-0384-4

24.	Sterne JAC, Savović J, Page MJ, Elbers RG, Blencowe NS, Boutron I, et al. RoB 2: A revised tool for assessing risk of bias in randomised trials. The BMJ [Internet]. 2019 [cited 2022 Sep 30];366:l4898. Available from: https://jhu.pure.elsevier.com/en/publications/rob-2-a-revised-tool-for-assessing-risk-of-bias-in-randomised-tri

25.	Sterne JA, Hernán MA, Reeves BC, Savović J, Berkman ND, Viswanathan M, et al. ROBINS-I: a tool for assessing risk of bias in non-randomised studies of interventions. BMJ [Internet]. 2016 Oct 12 [cited 2022 Sep 30];355. Available from: https://www.bmj.com/content/355/bmj.i4919

26.	Guyatt GH, Oxman AD, Vist GE, Kunz R, Falck-Ytter Y, Alonso-Coello P, et al. GRADE: an emerging consensus on rating quality of evidence and strength of recommendations. BMJ [Internet]. 2008 Apr 24 [cited 2022 Aug 3];336(7650):924–6. Available from: https://www.bmj.com/content/336/7650/924

27.	Laurienzo CE, Magnabosco WJ, Jabur F, Faria EF, Gameiro MO, Sarri AJ, et al. Pelvic floor muscle training and electrical stimulation as rehabilitation after radical prostatectomy: a randomized controlled trial. J Phys Ther Sci [Internet]. 2018 [cited 2022 Aug 2];30(6):825–31. Available from: https://pubmed.ncbi.nlm.nih.gov/29950773/

28.	Wille S, Sobottka A, Heidenreich A, Hofmann R. Pelvic floor exercises, electrical stimulation and biofeedback after radical prostatectomy: results of a prospective randomized trial. J Urol [Internet]. 2003 Aug 1 [cited 2022 Aug 2];170(2 Pt 1):490–3. Available from: https://pubmed.ncbi.nlm.nih.gov/12853806/

29.	Ribeiro LHS, Prota C, Gomes CM, de Bessa J, Boldarine MP, Dall’Oglio MF, et al. Long-term effect of early postoperative pelvic floor biofeedback on continence in men undergoing radical prostatectomy: a prospective, randomized, controlled trial. J Urol [Internet]. 2010 Sep [cited 2022 Aug 2];184(3):1034–9. Available from: https://pubmed.ncbi.nlm.nih.gov/20643454/

30.	Rajkowska-Labon E, Bakuła S, Kucharzewski M, Śliwiński Z. Efficacy of physiotherapy for urinary incontinence following prostate cancer surgery. Biomed Res Int [Internet]. 2014 [cited 2022 Aug 2];2014. Available from: https://pubmed.ncbi.nlm.nih.gov/24868546/

31.	Oh JJ, Kim JK, Lee H, Lee S, Jin Jeong S, Kyu Hong S, et al. Effect of personalized extracorporeal biofeedback device for pelvic floor muscle training on urinary incontinence after robot-assisted radical prostatectomy: A randomized controlled trial. Neurourol Urodyn [Internet]. 2019 Feb 1 [cited 2022 Aug 2];39(2):674–81. Available from: https://pubmed.ncbi.nlm.nih.gov/31793032/

32.	Moore KN, Griffiths D, Hughton A. Urinary incontinence after radical prostatectomy: a randomized controlled trial comparing pelvic muscle exercises with or without electrical stimulation. BJU Int [Internet]. 1999 [cited 2022 Aug 2];83(1):57–65. Available from: https://pubmed.ncbi.nlm.nih.gov/10233453/

33.	Marchiori D, Bertaccini A, Manferrari F, Ferri C, Martorana G. Pelvic Floor Rehabilitation for Continence Recovery after Radical Prostatectomy: Role of a Personal Training Re-Educational Program. Anticancer Res. 2010;30(2).

34.	Floratos DL, Sonke GS, Rapidou CA, Alivizatos GJ, Deliveliotis C, Constantinides CA, et al. Biofeedback vs verbal feedback as learning tools for pelvic muscle exercises in the early management of urinary incontinence after radical prostatectomy. BJU Int [Internet]. 2002 [cited 2022 Aug 2];89(7):714–9. Available from: https://pubmed.ncbi.nlm.nih.gov/11966630/

35.	de Santana e Santos NA, Saintrain MV de L, Regadas RP, da Silveira RA, de Menezes FJC. Assessment of Physical Therapy Strategies for Recovery of Urinary Continence after Prostatectomy. Asian Pac J Cancer Prev [Internet]. 2017 [cited 2022 Aug 2];18(1):81. Available from: /pmc/articles/PMC5563123/

36.	An D, Wang J, Zhang F, Wu J, Jing H, Gao Y, et al. Effects of Biofeedback Combined With Pilates Training on Post-prostatectomy Incontinence. Urology. 2021 Sep 1;155:152–9.

37.	Ahmed MT, Mohammed AH, Amansour A. Effect of pelvic floor electrical stimulation and biofeedback on the recovery of urinary continence after radical prostatectomy. Turkiye Fiziksel Tip ve Rehabilitasyon Dergisi. 2012;58(3):170–6.

38.	Sciarra A, Viscuso P, Arditi A, Mariotti G, de Berardinis E, di Pierro GB, et al. A biofeedback-guided programme or pelvic floor muscle electric stimulation can improve early recovery of urinary continence after radical prostatectomy: A meta-analysis and systematic review. Int J Clin Pract [Internet]. 2021 Oct 1 [cited 2022 Aug 4];75(10). Available from: https://pubmed.ncbi.nlm.nih.gov/33811418/

39.	Geraerts I, van Poppel H, Devoogdt N, Joniau S, van Cleynenbreugel B, de Groef A, et al. Influence of preoperative and postoperative pelvic floor muscle training (PFMT) compared with postoperative PFMT on urinary incontinence after radical prostatectomy: a randomized controlled trial. Eur Urol [Internet]. 2013 Nov [cited 2022 Aug 4];64(5):766–72. Available from: https://pubmed.ncbi.nlm.nih.gov/23357349/

40.	Strączyńska A, Weber-Rajek M, Strojek K, Piekorz Z, Styczyńska H, Goch A, et al. The Impact Of Pelvic Floor Muscle Training On Urinary Incontinence In Men After Radical Prostatectomy (RP) – A Systematic Review. Clin Interv Aging [Internet]. 2019 [cited 2022 Aug 4];14:1997. Available from: /pmc/articles/PMC6858802/

41.	Kannan P, Winser SJ, Fung B, Cheing G. Effectiveness of Pelvic Floor Muscle Training Alone and in Combination With Biofeedback, Electrical Stimulation, or Both Compared to Control for Urinary Incontinence in Men Following Prostatectomy: Systematic Review and Meta-Analysis. Phys Ther [Internet]. 2018 Nov 1 [cited 2022 Aug 5];98(11):932–45. Available from: https://pubmed.ncbi.nlm.nih.gov/30137629/

42.	Moore KN, Valiquette L, Chetner MP, Byrniak S, Herbison GP. Return to continence after radical retropubic prostatectomy: a randomized trial of verbal and written instructions versus therapist-directed pelvic floor muscle therapy. Urology [Internet]. 2008 Dec [cited 2022 Aug 4];72(6):1280–6. Available from: https://pubmed.ncbi.nlm.nih.gov/18384853/

43.	Dubbelman Y, Groen J, Wildhagen M, Rikken B, Bosch R. The recovery of urinary continence after radical retropubic prostatectomy: a randomized trial comparing the effect of physiotherapist-guided pelvic floor muscle exercises with guidance by an instruction folder only. BJU Int [Internet]. 2010 [cited 2022 Aug 4];106(4):515–22. Available from: https://pubmed.ncbi.nlm.nih.gov/20201841/

44.	Hall LM, Aljuraifani R, Hodges PW. Design of programs to train pelvic floor muscles in men with urinary dysfunction: Systematic review. Neurourol Urodyn [Internet]. 2018 Sep 1 [cited 2022 Aug 5];37(7):2053–87. Available from: https://pubmed.ncbi.nlm.nih.gov/29687914/

45.	Chughtai B, Lee R, Sandhu J, Te A, Kaplan S. Conservative Treatment for Postprostatectomy Incontinence. Rev Urol [Internet]. 2013 [cited 2022 Aug 5];15(2):61. Available from: /pmc/articles/PMC3784969/

46.	García-Sánchez E, Ávila-Gandía V, López-Román J, Martínez-Rodríguez A, Rubio-Arias J. What Pelvic Floor Muscle Training Load is Optimal in Minimizing Urine Loss in Women with Stress Urinary Incontinence? A Systematic Review and Meta-Analysis. Int J Environ Res Public Health [Internet]. 2019 Nov 2 [cited 2022 Aug 5];16(22). Available from: /pmc/articles/PMC6887794/

47.	Stafford RE, Ashton-Miller JA, Constantinou C, Coughlin G, Lutton NJ, Hodges PW. Pattern of activation of pelvic floor muscles in men differs with verbal instructions. Neurourol Urodyn [Internet]. 2016 Apr 1 [cited 2022 Aug 4];35(4):457–63. Available from: https://pubmed.ncbi.nlm.nih.gov/25727781/

48.	Kruger J, Budgett D, Goodman J, Kari B. Can you train the pelvic floor muscles by contracting other related muscles? Neurourol Urodyn [Internet]. 2019 Feb 1 [cited 2022 Aug 4];38(2):677–83. Available from: https://pubmed.ncbi.nlm.nih.gov/30592502/

49.	Jacomo RH, Nascimento TR, Lucena da Siva M, Salata MC, Alves AT, da Cruz PRC, et al. Exercise regimens other than pelvic floor muscle training cannot increase pelvic muscle strength-a systematic review. J Bodyw Mov Ther [Internet]. 2020 Oct 1 [cited 2022 Aug 5];24(4):568–74. Available from: https://pubmed.ncbi.nlm.nih.gov/33218562/

50.	Wang C, Li J, Wan X, Wang X, Kane RL, Wang K. Effects of stigma on Chinese women’s attitudes towards seeking treatment for urinary incontinence. J Clin Nurs [Internet]. 2015 Apr 1 [cited 2022 Oct 6];24(7–8):1112–21. Available from: https://pubmed.ncbi.nlm.nih.gov/25422008/

Figure legends:

Figure 1: PRISMA 2020 flow diagram.

Figure 2: Risk of bias assessment of the included studies.

Figure 3: Meta-analysis for the primary outcome (1h pad test) at week 4.

Figure 4: Meta-analysis for the primary outcome (24h pad test) at week 12.

Table legends:

Table 1: Characteristics of the studies included.

Table 2: Primary outcome (pad test) of the studies included.

Table 3: Secondary outcome (health-related quality of life) of the studies included.

Table 4: GRADE approach assessment.

Supplementary materials legends:

Supplementary material 1: Database research strings.
